# 

**Published:** 1879-10

**Authors:** 


### CONTENTS.

### COMMUNICATIONS.

Review of the Transactions of the Dental Association of Maryland and District of Columbia. *By Martin P. Scott, M. D. of Baltimore* 405
Review of the Conservative Treatment of the Dental Pulp. *By Louis Jack, D. D. S. Philadelphia*..........409
Dental Caries. *By C. N. Pieroe, D. D. S., Philadelphia*..........421

### PROCEEDINGS OF SOCIETIES.

Minutes of the Ninth Annual Meeting of the Kentucky State Dental Association..........425

### MISCELLANEOUS.

Disinfectants, and How to Use Them..........430
Aphtha of the Gums. *Translated from the French by Dr. David*..........433
The Internal and External Action of Thymic Acid and Thymate of Soda..........437
Correspondence..........439

### EDITORIAL.

Obituary..........441
American Academy of Dental Science..........441
Bibliographical..........445
Absence..........448
Obituary..........448

